# Hinge-FM2I: an approach using image inpainting for interpolating missing data in univariate time series

**DOI:** 10.1038/s41598-025-86382-4

**Published:** 2025-02-13

**Authors:** Saad Noufel, Nadir Maaroufi, Mehdi Najib, Mohamed Bakhouya

**Affiliations:** TICLab and LERMA Lab, College of Engineering and Architecture, International University of Rabat, 11000 Sala Al Jadida, Morocco

**Keywords:** Univariate time series, Missing data imputation, Interpolation, Image inpainting, Mathematics and computing, Computer science

## Abstract

Accurate time series forecasts are crucial for various applications, such as traffic management, electricity consumption, and healthcare. However, limitations in models and data quality can significantly impact forecasts’ accuracy. One common issue with data quality is the absence of data points, referred to as missing data values. It is often caused by sensor malfunctions, equipment failures, or human errors. This paper proposes Hinge-FM2I, a novel method for handling missing data values in univariate time series data. Hinge-FM2I builds upon the strengths of the Forecasting Method by Image Inpainting (FM2I). FM2I has proven effective, but selecting the most accurate forecasts remains a challenge. To overcome this issue, we proposed a selection algorithm. Inspired by door hinges, Hinge-FM2I drops a data point either before or after the gap (left/right-hinge), then uses FM2I for imputation. In fact, it selects the imputed gap based on the lowest error of the dropped data point. Hinge-FM2I was evaluated on a comprehensive sample composed of 1356 time series. These latter are extracted from the M3 competition benchmark dataset, with missing value rates ranging from 3.57 to 28.57%. Experimental results demonstrate that Hinge-FM2I significantly outperforms established methods such as linear/spline interpolation, K-Nearest Neighbors, and ARIMA. Notably, Hinge-FM2I achieves an average Symmetric Mean Absolute Percentage Error score of 5.6% for small gaps and up to 10% for larger ones. These findings highlight the effectiveness of Hinge-FM2I as a promising new method for addressing missing values in univariate time series data.

## Introduction

In today’s data-driven world, forecasting future values of a time series when real-time data points come progressively through time, called online time series forecasting^1^, has become a necessary tool for extracting useful insights from the continuous data flows. This type of forecasting is becoming increasingly important across a variety of domains, such as traffic management^2^, electricity consumption^3^, healthcare^4^, weather data^5,6^, transportation^7^, energy management^8^, and water-related disease prediction^9^. Recently, the increasing adaptation of artificial intelligence (AI) has shown its potential in handling this type of forecasting. In fact, AI-based models must be continuously updated as new data observations become available. This allows the model to adapt to changes in the underlying time series, while making more accurate forecasts. However, the continuous adaptation of these models to new data introduces additional complexities. AI-based models must not only process real-time data streams but also contend with concept drift, where the statistical properties of the target variable change over time in unforeseen ways^10^, potentially degrading prediction accuracy. Furthermore, the use of real-time data presents its own set of challenges, such as data availability^11^ and data quality^12^. Some issues related to real-time data quality include the potential to introduce noise, anomalies, or missing data^13^. This latter, also referred to as missing values, occurs when there is no data stored for certain variables^14^. In other terms, missing data refers to the absence of observations that are expected to be present. Missing data can happen for various reasons. For instance, in the context of Internet of Things (IoT), missing data can occur due to various reasons, such as sensor malfunction, network failure, or data transmission errors^15^. While in the context of particulate air pollutant, it can occur due to power supply failures, problems with air aspiration pumps, or electronic processing malfunction^16^.

A large gap of missing data, of size *T*, is an interval $[x_i:x_{i + T - 1}[$ containing consecutive missing values. It poses significant challenges for online forecasting models as they introduce substantial information loss, making it difficult to accurately predict future values^17^. To address this challenge, two main approaches are commonly employed to handle large gaps of missing data values: *deletion* or *imputation*. The first one simply involves ignoring observations with missing values. Deletion approach consists of two techniques, listwise deletion, and pairwise deletion. The first discards all observations with missing values for any variable of interest, while the second does not exclude the entire unit, but uses as much data as possible. However, despite the low implementation complexity of the deletion approach, it significantly reduces the size of the dataset, which might lead to a loss of significant information^18^. The imputation approach estimates the missing values based on available information and underlying patterns. Multiple techniques, such as mean, median, last observation carried forward (LOCF), and interpolation techniques, are frequently used to fill in missing data in univariate datasets^19–21^. While these methods can handle a few consecutive missing values or short data gaps, they are not well-suited for dealing with multiple types of data gaps, particularly large ones^22^. Despite that common approaches, like deletion and imputation, have limitations in handling large data gaps, some researchers have proposed leveraging data from nearby sensors or devices experiencing similar conditions. For instance^23^, proposed a method combining multi-agent systems and IoT to impute missing values by using mobile intermittent IoT devices and local computations, especially in areas where sensors perceive analogous dynamics, e.g., temperature sensors in an area might provide values with similar fluctuations over time because of shared weather patterns. In fact, this spatial approach aims to better estimate missing information in large-scale data analysis with complex, multiple data gaps.

In this research paper, we propose a novel imputation method to address large gaps of missing data. The technique begins by identifying the gap and dropping/storing one known value, either before or after the gap. The resulting time series is then fed into a forecasting method based on FM2I^24^. This latter mainly transforms the time series into an image, applies a mask, and then employs a patch-based image inpainting approach to fill the masked region. Once the image is inpainted, it is converted again to a matrix, and a selection algorithm is then applied to select the most accurate forecasts. Unlike the original FM2I, which focuses on forecasting (i.e., extrapolation), our objective is to impute missing data through interpolation. To adapt FM2I for this purpose, we propose a simple shift in the mask position, allowing us to leverage its image inpainting capabilities for interpolation. We introduce a selection algorithm, named Hinge-FM2I, which compares the dropped value with the generated value and then selects the sequence with the minimal difference error. To showcase the efficiency of our approach, an extensive evaluation was conducted on a diverse sample, which is extracted from the M3 benchmark dataset^25^. The obtained results demonstrate that Hinge-FM2I significantly outperforms existing techniques in imputing large gaps of missing data.

In summary, the main general contributions of this work are two fold:

- Introduce Hinge-FM2I to impute large gaps of missing data based on the forecasting by image inpainting.
- Extensive experiments are conducted to evaluate the proposed method and compare it against different commonly used imputation methods as well as real recorded observations (ground truth).

The remainder of this paper is structured as follows. Section 2 provides a comprehensive state-of-the-art review of the methods handling missing data in univariate time series. Section 3 introduces Hinge-FM2I for imputing large gaps of missing data in univariate time series. Section 4 reveals our results, showcasing the effectiveness of our approach compared to existing techniques. Section 5 summarizes our findings, while highlighting perspectives.

## Related work

Real-time forecasting refers to the process of predicting future values of a time series as new data becomes available. This approach involves continuously updating the model with the latest observations and using it to make predictions for the next time horizon^26^. It holds utmost importance in today’s data-driven world, but is faced with data quality problems. Among these problems, the existence of missing data. It causes the reliability and accuracy of forecasting models to heavily drop. Large gaps of missing data can be identified as sequences of consecutive data points that are missing. The size of the gap is determined by the number of consecutive missing data points. Beginning at a specific point, $x_t$, and extending to $x_{t+T-1}$, where *T* represents the size of this gap. The missing data in these gaps can be classified into three types, missing completely at random (MCAR), missing at random (MAR) and Not missing at random (NMAR)^27^. It is essential to know why data is missing in order to choose a good method to deal with those gaps. Understanding the reasons behind the missing data can help pick the right imputation technique^28^. But in reality, figuring out those reasons is frequently difficult, particularly when there is absolutely no information available about the missing data, or when the missing data follows a complicated pattern^29^. To the best of our knowledge, most current research works focus on the three defined missing data types. The following sections presents the employed methods to deal with missing data in univariate time series.

Missing data in univariate time series can be dealt following two main approaches: *deletion* or *imputation*. On one hand, the deletion approach entirely removes observations with missing values from the datasets. While this approach is straightforward and easy to implement, it often leads to substantial information loss, especially when large gaps of missing data are present^18^. On the other hand, imputation techniques are employed to estimate the missing values based on the available information and the underlying patterns in the time series. Imputation offers the advantage of retaining the full datasets, thereby preserving valuable information while maintaining the continuity of the time series^18^. However, the extra uncertainty added by the imputation process itself is usually not taken into account. This means the calculated variance of the estimated relationships in the data is typically underestimated and appears smaller than it actually is. Another key issue with imputation methods, especially the simpler types, is that they can produce biased or inaccurate estimates of the associations or connections between variables in the data^30^.

**Figure 1:**
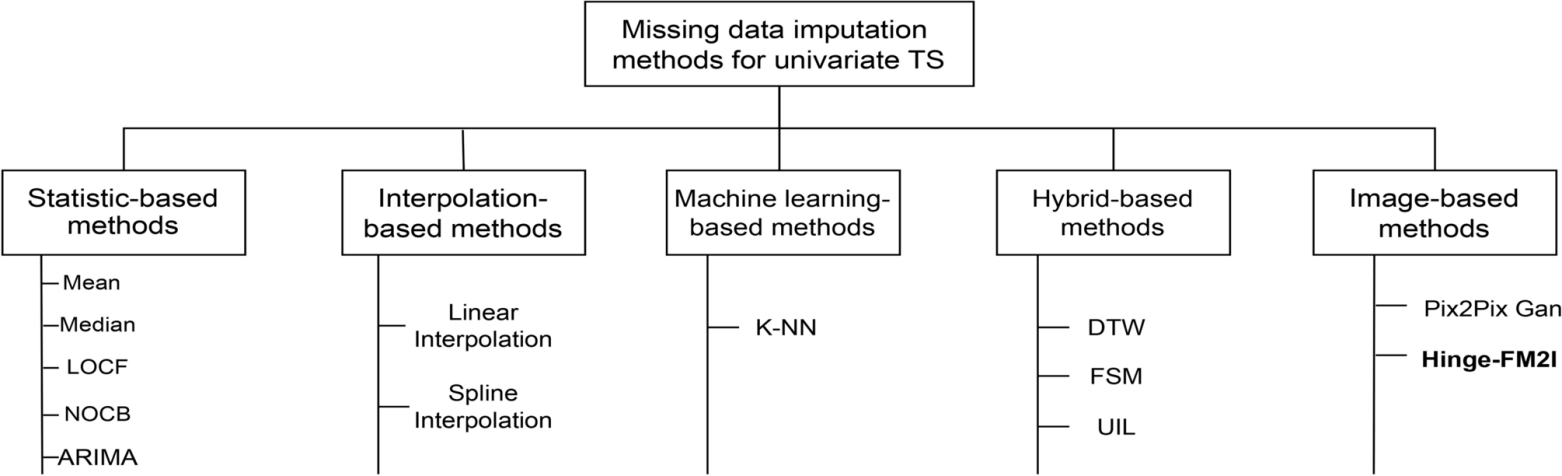
Comprehensive overview of missing data imputation methods.

In the past decade, several methods have been proposed to deal wiht missing data in univariate time series. These methods can be classified into five sub-categories, as depicted in Fig. 1: *Statistical-based*, *Interpolation-based*, *Machine learning-based*, *Hybrid-based*, and *Image-based*. Firstly, statistical-based methods estimate and replace missing values with statistical measures, such as mean, median, LOCF, next observation carried backward (NOCB), or ARIMA^31^. While these methods are simple and easy to implement with a fast computation time. However, these methods may not work well when a large amount of data is missing^32^. For instance, these methods underestimate the variance, ignores the correlation between the features, thus often leads to poor imputation^33^. Secondly, interpolation-based methods such as, linear and spline interpolation, creates a continuous function that approximates the relationship between the observed data points. On one hand, linear interpolation constructs a straight line between two known data points and estimates the missing value based on this line. On the other hand, spline interpolation uses piecewise polynomial functions to create a smooth curve that passes through the known data points, providing a more accurate estimation of missing values^34^. Even though it is easier to implement, linear interpolation assumes a linear relationship between data points, which might not always reflect the true underlying patterns in the time series data, meanwhile, spline interpolation does not consider the underlying structure of the data, which can result in inaccurate imputations^35^. Thirdly, machine learning-based methods deal with missing data by creating a predictive model to estimate values that will substitute the missing items. By learning patterns and relationships from the available data, it allows them to make predictions and fill in missing values based on the existing information in the dataset. For instance, k-Nearest Neighbor (kNN)^36^ is widely used to impute missing data in univariate time series.

While traditional imputation techniques, like deletion and interpolation methods, have been widely used, they often fall short when dealing with complex patterns or large gaps in univariate time series data. To address these limitations, researchers have proposed more advanced state-of-the-art hybrid-based methods. One class of methods focuses on aligning and matching the observed and missing portions of the time series. For instance, the authors in^37^ proposed, Dynamic Time Warping (DTW), an imputation technique that compares two time series by aligning to minimize the differences between corresponding points. DTW-based imputation works by finding the best alignment between the observed and missing parts of the time series, and then using this alignment to estimate the missing value. However, the quality of the imputed values depends on the accuracy of the alignment. Similarly^38^, introduced Full Subsequence Matching (FSM), which identifies the most similar sub-sequence from historical data and adapts it to fit the missing part. Nevertheless, FSM may not perform well if there are too many missing values or if historical patterns are not representative. Another approach involves combining univariate and multivariate imputation techniques, as proposed in^39^ for marine machinery systems. While this framework shows promise in that domain, potentially its applicability and limitations in other contexts are not extensively discussed, limiting the generalization of the findings. In their work^40^, proposed a univariate imputation method (UIM) specialized for wastewater treatment processes. The UIM approach consists of three main steps. Firstly, missing gaps in the data are initially filled using linear interpolation. Secondly, the UIM applies a seasonal trend decomposition procedure based on locally estimated scatterplot smoothing (loess), also known as STL. After decomposing the time series, the trend and remainder components are imputed separately using support vector regression (SVR), while the seasonal component is imputed through self-similarity decomposition (SSD). In the final step, the imputed components are recombined by summing them, yielding the imputed time series.

Recently, forecasting by image inpainting (FM2I)^24^ was proposed to extrapolate univariate time series. But when it comes to interpolating missing data in univariate time series, to the best of our knowledge, the only approach that tackles this issue was proposed in^41^. They proposed an approach, using a conditional generative adversarial network (Pix2Pix GAN), to impute missing data by transforming time series into images. However, the performance of this method may depend on the selected network parameters. In this direction, the present research work introduces, based on image inpainting principles, the Hinge-FM2I for imputing univariate time series. Table 1 resumes the above mentioned approaches.

**Table 1:** Imputation-based approaches for univariate time series.

Classes	Approaches	Gap size	Computational time	Implementation complexity
Statistic-based	Mean^31^	Small	Low	Easy
	Median^31^	Small	Low	Easy
	LOCF^31^	Small	Low	Easy
	NOCB^31^	Small	Low	Easy
	ARIMA^31^	Small	Low	Easy
Interpolation-based	Linear^34^	Large	Low	Easy
	Spline^34^	Large	High	Easy
ML-based	K-NN^36^	Small	High	Medium
Hybrid-based	DTW^37^	Large	High	Easy
	FSM^38^	Large	High	Medium
	UIM^40^	Large	High	Medium
Image-based	Pix2Pix Gan^41^	Large	High	Hard
	Hinge-FM2I	Large	High	Medium

## Hinge-FM2I approach

**Figure 2:**
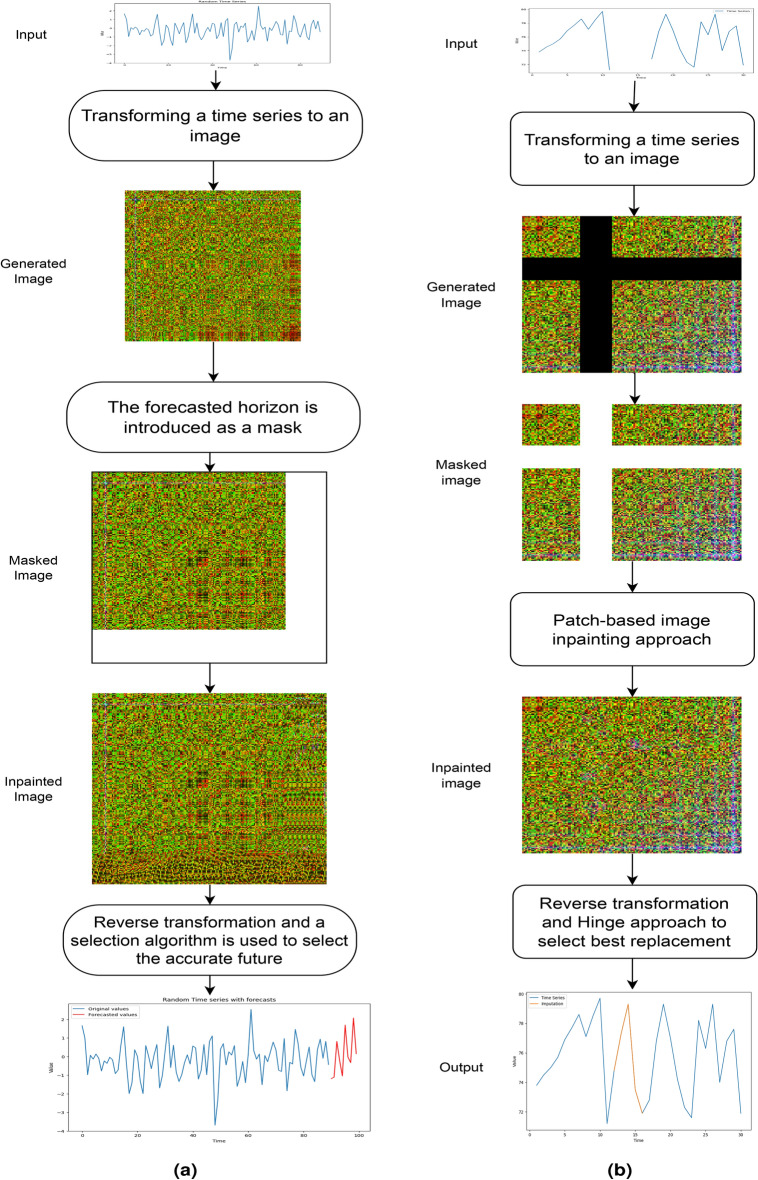
(**a**) FM2I and (**b**) Hinge-FM2I framework.

Dealing with missing data in univariate time series is a significant challenge, since missing data make it difficult to accurately forecast future values. The above-mentioned methods may fail to capture complex underlying patterns, leading to inaccurate estimates or loss of valuable information, especially when dealing with large gaps of missing data. To address these limitations, we propose Hinge-FM2I, a new method for filling in missing values in univariate time series data. Hinge-FM2I is an extension of FM2I^24^. It is worth noting that, FM2I algorithm can be structured into three steps as depicted in Fig. 2a. The first step consists of all the steps required for preparing the time series. It consists of rescaling to either $[-1,1]$ or [0, 1]. Once a rescaled time series is calculated, noted $res\_TS$, it is transformed to its corresponding image. The second step consists of extrapolating and inpainting the generated images using a patch-based algorithm. Note that the mask correspond to the forecasting horizon. In the third and last step, the reverse process is applied in order to both extract the forecast values from the image and reverse all the scaling steps.

Although experiments showed great results in terms of forecasting accuracy, FM2I has some limitations. First, the patch-based image inpainting approach uses existing data to choose the most suitable pixel to inpaint. However, with large gaps of missing values in the time series data, the generated image is affected, likely influencing the patch-based approach to introduce mismatch error. Second, choosing the accurate forecast, for both extrapolation and interpolation, requires defining a new selection algorithm. Hinge-FM2I introduces a selection technique inspired from door hinges principles, aiming to select the best sequence from a matrix of possible imputation sequences. A general overview of the Hinge-FM2I is depicted in Fig. 2b, while its pseudo-code algorithm, which consists of seven main steps, is detailed in Algorithm 1.

**Algorithm 1:**
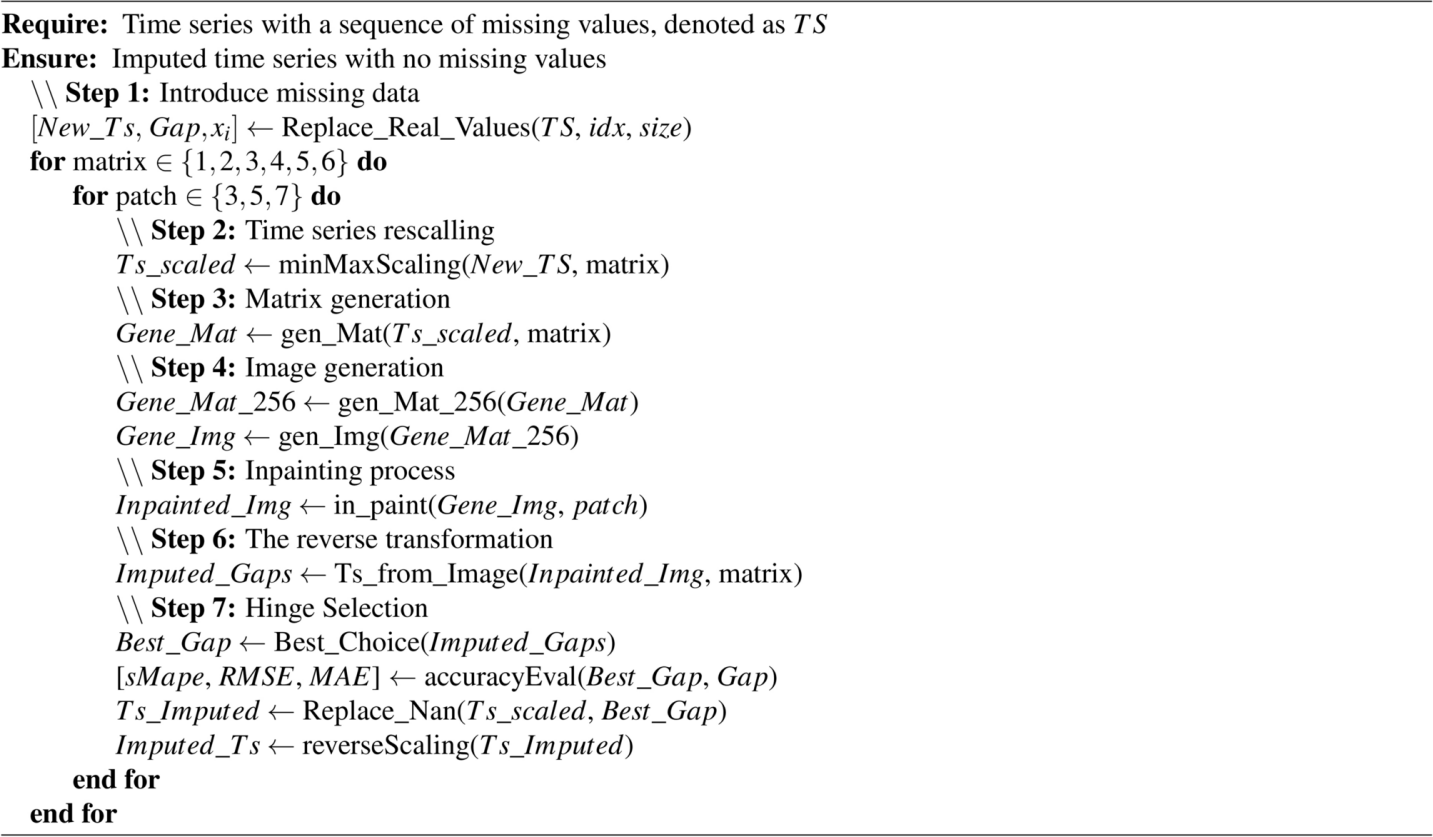
Hinge-FM2I Algorithm for Time Series Imputation

The *first step* addresses the need for simulated (i.e., randomly generated) missing data, required to evaluate imputation performance. In our experimental framework, all missing data are randomly introduced to ensure controlled testing conditions while enabling accurate evaluation of the imputation-based methods. The original datasets were complete time series, allowing us to establish ground truth values for subsequent accuracy assessments. The missing data simulation process followed a random introduction approach, similarly to the standardized protocol used in^37,40^. The gaps lengths are set to 5, 10, and 20 time steps (respectively 5%, 10%, 15%, and 20%) for the first (respectively second) experiment. These gaps were inserted at randomly selected positions within the time series. This randomization was implemented using a probabilistic mechanism that ensured an unbiased distribution of gaps throughout the dataset. For each gap size, we employed a uniform random number generator to determine the starting position of the gap. The values at these selected positions were systematically removed and stored separately for later comparison with the imputed values, enabling precise measurement of the algorithm’s performance.

In the *second step*, the time series undergoes min-max normalization, transforming all values to a scale between 0 and 1. This standardization is accomplished by first subtracting the minimum value from each data point, then dividing by the range (maximum minus minimum), ensuring consistent data scaling across the entire series. The *third step* follows the methodology outlined in^24^. The normalized one-dimensional time series is converted into a two-dimensional matrix representation. There are six possible matrices, which can be used to capture both temporal patterns and correlation information within the data. In the *fourth step*, a one-to-one function is employed to encode the scaled matrix values into RGB color components. Each value is mapped to a unique combination of Red, Green, and Blue values (ranging from 0 to 255), creating a reliable color encoding scheme, representing up to $256^3$ distinct values.

The *fifth step* consists of using the patch-based algorithm^42^, which takes a geometric approach to image inpainting, focusing on both the structure and texture of the image. The algorithm prioritizes the filling order based on two key terms: a confidence and a data term. The confidence term measures the reliability of the pixel values surrounding the target area, while the data term evaluates the strength and orientation of isophotes (lines of equal intensity) intersecting the boundary of the target region. The priority value for each patch is calculated as the product of these two terms, ensuring that strong linear structures are propagated first while maintaining the overall texture coherence. For instance, if a strong edge meets the boundary of the missing region, patches along that edge receives higher priority, allowing the algorithm to maintain structural continuity. Once the priority is determined, the algorithm searches for the most similar patch in the source region (the known part of the image) to fill in the target area. This similarity is computed using the sum of squared differences metric that considers the already filled pixels in the patch. The matched patch is then used to fill in the missing values of the image. This process is repeated iteratively until the entire target region is filled. Once the image is inpainted, the *sixth step* reverses the image transformation process to extract all possible imputed values, resulting in a matrix containing potential imputation sequences for the missing data points.

**Figure 3:**
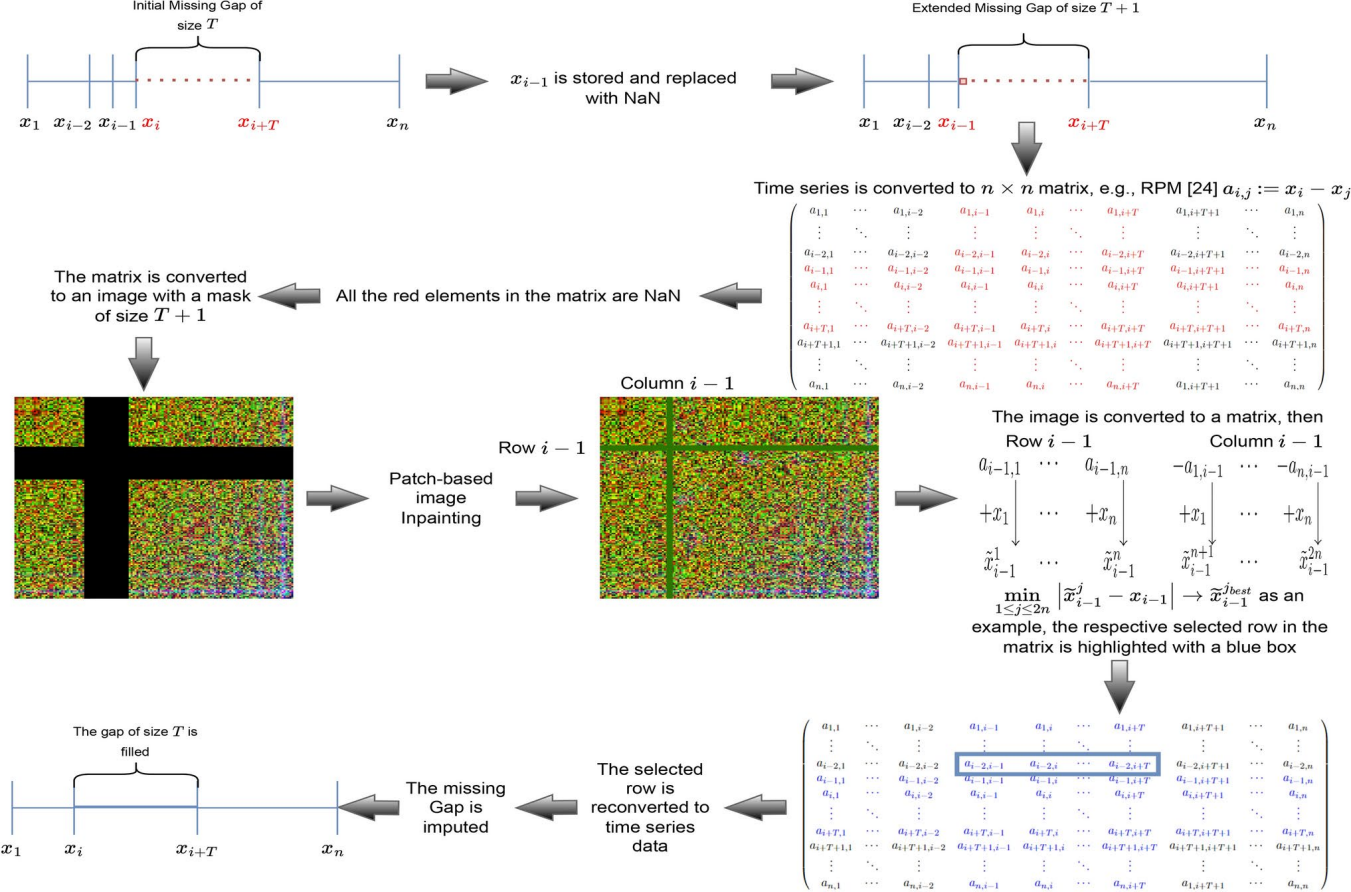
The Hinge process for selecting best imputation.

The *seventh step* consists of applying the hinge algorithm to select the best imputation from the resulting matrix. The process starts by comparing the real value of $x_{i-1}$, already stored in *first step*, against the generated values $\tilde{x}_{i-1}$ using the absolute difference. Therefore, the imputed data are selected based on the minimum of the absolute difference. For instance, a gap of size *T* starting from data point denoted $x_{i}$ to $x_{i+T}$, the hinge algorithm will store the data point $x_{i-1}$ and change its value to NaN. Once the inpainting process is completed, the image is converted back to a matrix, then the absolute differences are calculated between $x_{i-1}$ and all its estimated values $\tilde{x}_{i-1}$. Then the row or column with the minimum absolute difference corresponds to the best selection to impute the gap. The time series is then imputed and reversed to the real scale. More detailed overview of the hinge process is graphically described in Fig. 3.

## Experimental results

### Datasets, methods and metrics

In order to assess the effectiveness of Hinge-FM2I, we have taken a sample from the M3 competition^25^. We mainly took into consideration time series with a minimum of 70 observations. The sample dataset includes 1356 time series, which are categorized according to their types (e.g., industry, finance) and time intervals (e.g., Monthly, quarterly, yearly) as shown in Table 2. We also used two public waste water treatment process public datasets to compare against state-of-the-art approaches, the first dataset can be found in (UCI) and it is a collection of waste water treatment plant in Spain, while the other is simply $NH_4$ concentration and can be obtained from imputeTS package in R software (Table 3).

**Table 2:** M3-competition dataset, types and description^25^.

Interval	Micro	Industry	Macro	Finance	Demog	Other	Total
Yearly	146	102	83	58	245	11	645
Quarterly	204	83	336	76	57	0	756
Monthly	474	334	312	145	111	52	1428
Other	4	0	0	29	0	141	174
Total	828	519	731	308	413	204	3003

**Table 3:** Waste water and NH4 datasets description.

No.	Time series name	Length	Maximum	Minimum	Mean
1	Input pH to plant (PH.E)	200	8.5	7.3	7.83
2	Input sediments to primary settler (SED.P)	200	46.0	1.0	5.12
3	Output suspended solids (SS.S)	200	131.0	8.0	20.91
4	Output sediments (SED.S)	200	3.5	0.0	0.04
5	Performance input biological demand of oxygen in primary settler (RD.DBO.P)	200	79.1	0.6	39.13
6	NH4 concentration (NH4)	4552	48.2	0.7	12.4

For the sake of evaluating the performance of the proposed method. Firstly, we compared with nine other existing methods for univariate time series (mean, median, LOCF, NOCB, LI, SI, KNN, ARIMA, DTWBI). All these methods are implemented using Python language, except the DTWBI, which exists as a package in R language. The spline interpolation method employed a cubic polynomial function, whereas for the KNN approach, a grid search was performed to determine the optimal number of clusters. The parametres of the above mentionned approaches are described in the Table 4. To eliminate the uncertainty of imputation results, the experiments are repeated 10 times by randomly selecting the missing locations for each time series. Once the imputation of missing values is completed, we evaluate the performance of our method, and compare it with the above stated approaches based on three different metrics. First, Symmetric Mean Absolute Percentage Error (sMAPE) measures the percentage difference between predicted and actual values, taking into account the magnitude of the values being compared. It is expressed in percentage. Second, Root Mean Squared Error (RMSE) measures the average squared difference between predicted values and actual values, with the square root taken to obtain the final measure. Third, Mean Absolute Error (MAE) measures the average absolute difference between predicted values and actual values. It provides a straightforward measure of the average prediction error. Secondly, we compared our approach to two state-of-the-art methods^40,41^, and evaluated the results using two indicators RMSE and similarity (Sim).

**Table 4:** Comparison methods and their parameters.

Method	Parameters
Mean	None
Median	None
LOCF	None
NOCB	None
LI	None
SI	Order = 3
KNN	k = {3, 5, 7, 9, 11}, metric = Euclidean
ARIMA	Auto-ARIMA was used to determine the best (p,q,d)
DTWBI	Default parameters
Pix2Pix GAN	The same architecture as in^41^

### Results and discussion

Experiments have been conducted on a computer composed of the following characteristics, AMD Ryzen 7 3750H, 2.30 GHz, 12.00 GB RAM, x64 based processor, Windows 10. For the first experience, we have considered three missing data sequence lengths, 5, 10, and 20. These gaps sizes simulated a missing data ranging from 3.57% to 28.57%. On the other hand, for the second experience, we considered four missing ratio 5%, 10%, 15%, and 20%. The result and discussion section is divided into two subsections, first a numerical analysis of the imputation is presented and discussed, then a visual analysis is showcased.

**Figure 4:**
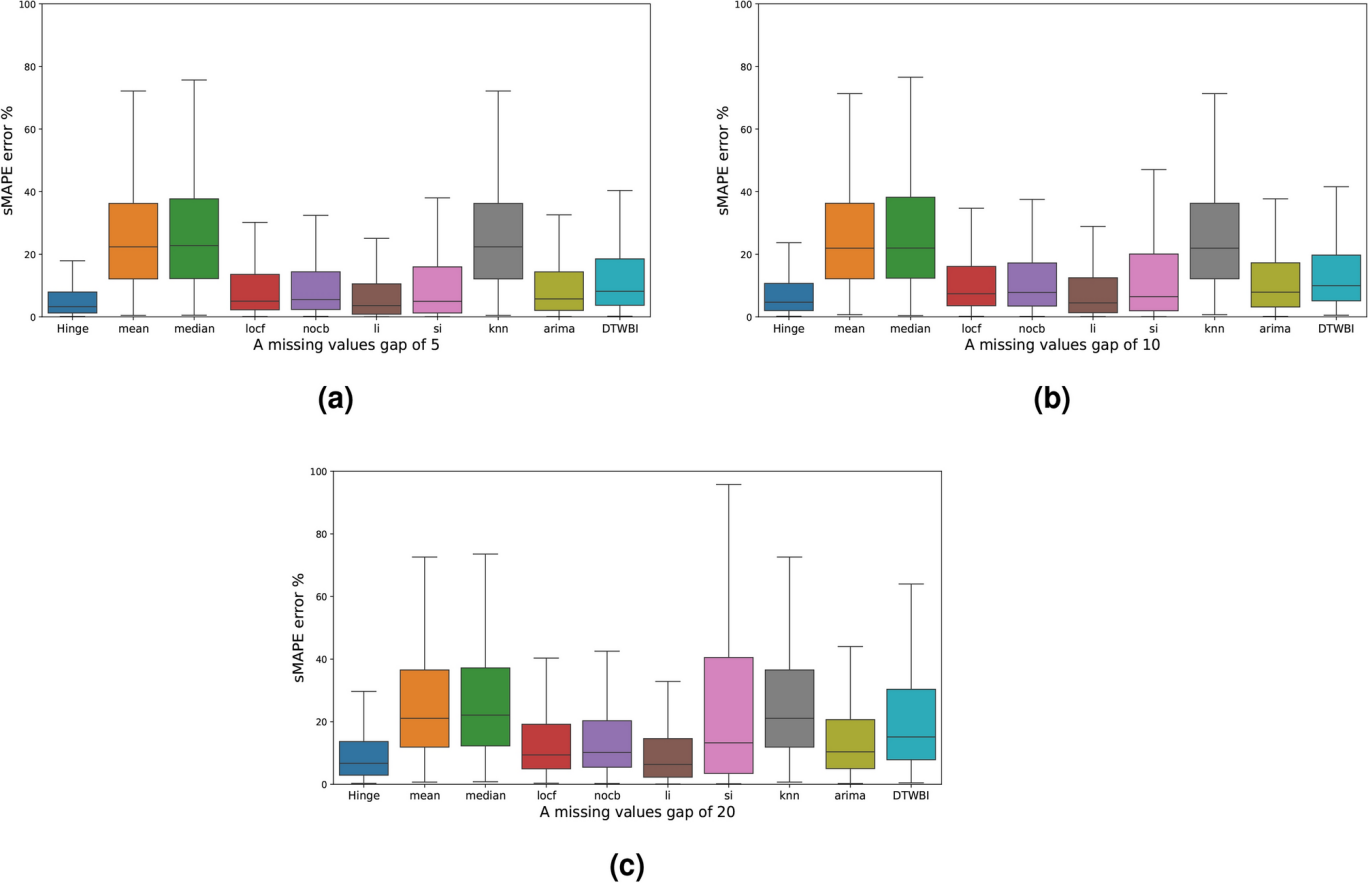
Box-plots of sMAPE for various imputation methods with different size of missing gaps.

In Table 5 the comparison, in terms of imputation accuracy, of the Hinge-FM2I against the commonly used methods. It showcases that Hinge-FM2I outperforms all the other methods in terms of all metrics. Apart from our method, only the LI method had good results in terms of sMAPE error. To better understand the effectiveness of the Hinge-FM2I, an analysis of Fig. 4a demonstrate the Hinge-FM2I approach compared to other methods. Its box plot shows a relatively low median, indicating consistently lower sMAPE values, which reflects higher prediction accuracy. In contrast, methods like “Mean” and “Knn” display larger interquartile ranges and higher median values, suggesting greater variability and lower accuracy. The “Median”, “Locf”, and “Nocb” approaches present moderate performance with a somewhat symmetrical distribution, but they do not match the accuracy and consistency of the Hinge-FM2I method. “LI” and “SI” methods exhibit significant variability and right skewness, indicating less reliable performance. The “Arima” method, while having a slightly left-skewed distribution, still shows a higher median than “Hinge-FM2I”. In Fig. 4b, the Hinge-FM2I approach again demonstrates superior performance based on the sMAPE metric. The box plot reveals that the Hinge-FM2I column has a centrally located median with a narrow interquartile range, indicating high accuracy and consistency. Other approaches show varied performance, with some methods exhibiting higher median sMAPE values and broader interquartile ranges, reflecting lower accuracy and greater variability. The overall distribution patterns of these methods highlight their inferiority compared to the Hinge-FM2I approach. The absence of significant outliers further underscores the robustness of the Hinge-FM2I method, making it the preferred choice for achieving lower sMAPE values and, consequently, better predictive performance. The evaluation of Fig. 4c through sMAPE metric results further solidifies the superiority of the Hinge-FM2I approach. The box plot for Hinge-FM2I displays a central median within a narrow interquartile range, signifying low sMAPE values and high prediction accuracy. In comparison, other methods show wider interquartile ranges and higher medians, indicative of lower accuracy and increased variability. Some methods, despite having a symmetrical distribution, still do not achieve the same level of performance as Hinge-FM2I. The presence of more significant variability and skewness in the alternative methods emphasizes the consistency and reliability of the proposed approach, time series with different features, such as trend or seasonality with various sizes, were taken as a sample and imputed. The results of this experiment are presented in Table 7. As shown in this table, the Hinge-FM2I method, in its current version, showcases great results, even across different types of time series. In summary, the Hinge-FM2I outperforms all the most considered methods in terms of accuracy. It is also worth nothing that, Hinge-FM2I takes into consideration neither the type of missing data nor the category of the time series.

We then evaluated the performance of our proposed method against the current state-of-the-art approaches from^40^, and^41^. As shown in Table 6, our method generally outperforms these methods. For instance, regarding the PH.E time series, our approach achieves a lower RMSE while maintaining equal similarity (Sim) values compared to the baseline. On the SED.P dataset, our method demonstrates better similarity results with equal RMSE performance. For the SS.S and SED.S time series, our approach yields higher similarity scores, though there is a trade-off in terms of increased RMSE values. With the RD.DBO.P data, we obtained lower RMSE but equivalent similarity to the previous work. Finally, on the $NH_4$ time series, our method improves RMSE performance at the cost of reduced similarity compared to the state-of-the-art methods. Overall, the results summarized in Table 6 highlight the competitive performance of our proposed approach across a range of time series benchmarks. While trade-offs between accuracy and similarity are observed for certain datasets, results obtained by our method consistently matches or exceeded state-of-the-art methods, in terms of accuracy (RMSE and Sim metrics).

**Table 5:** Comparison of imputation accuracy metrics.

Methods	Gap size of 5			Gap size of 10			Gap size of 20		
	sMAPE	RMSE	MAE	sMAPE	RMSE	MAE	sMAPE	RMSE	MAE
Left-Hinge	5.94	298.28	235.70	**8.23**	**461.18**	**349.31**	**10.32**	**602.87**	**458.23**
Right-Hinge	**5.69**	**285.56**	**223.69**	8.98	492.87	368.95	12.32	643.31	488.33
Mean	27.69	1392.29	1314.47	27.41	1374.47	1281.95	27.31	1399.82	1284.98
Median	28.65	1430.75	1350.45	28.48	1419.84	1324.00	28.47	1448.61	1331.56
LOCF	10.68	587.92	499.60	12.93	687.41	570.39	14.93	826.50	685.75
NOCB	11.99	629.39	531.70	13.76	706.33	587.85	16.16	846.82	699.50
LI	8.23	472.04	385.84	10.13	544.04	437.10	10.88	613.97	488.65
SI	14.97	677.85	586.02	19.69	915.10	785.89	31.23	1637.59	1398.90
KNN	27.69	1392.29	1314.47	27.41	1374.47	1281.95	27.31	1399.82	1284.98
ARIMA	16.90	803.17	711.87	20.48	897.30	776.31	25.57	1054.97	905.99
DTWBI	14.40	745.50	630.30	15.25	815.60	710.20	21.97	980.40	835.70

Significant values are in [bold].

**Table 6:** Comparison, using six time series, of Hinge-FM2I against state-of-the-arts methods.

Missing ratio	Method	PH.E		SED.P		SS.S		SED.S		RD.DBO.P		NH4	
		RMSE	Sim	RMSE	Sim	RMSE	Sim	RMSE	Sim	RMSE	Sim	RMSE	Sim
5%	Left-Hinge	**0.09**	**0.86**	**0.05**	**0.85**	0.83	0.86	0.36	**0.91**	**0.13**	0.81	**0.04**	0.73
	Right-Hinge	0.11	0.82	0.08	0.80	0.71	**0.90**	0.36	**0.91**	**0.13**	**0.82**	0.12	0.60
	UIM	0.13	0.82	**0.05**	0.79	**0.05**	0.76	**0.012**	0.75	0.16	0.80	0.08	**0.91**
	Pix2Pix GAN	**0.09**	0.49	0.13	0.1	0.07	0.48	0.013	0.65	0.20	0.75	0.42	0.60
10%	Left-Hinge	**0.07**	**0.85**	**0.05**	**0.84**	0.64	0.90	0.25	**0.95**	0.20	0.80	**0.03**	0.78
	Right-Hinge	0.16	0.80	0.06	0.81	0.56	**0.91**	0.26	**0.95**	**0.13**	**0.83**	0.11	0.60
	UIM	0.14	**0.85**	**0.05**	0.82	**0.08**	0.82	**0.010**	0.82	0.19	**0.83**	0.102	**0.89**
	Pix2Pix GAN	0.08	0.62	0.09	0.73	0.09	0.62	0.015	0.60	0.21	0.78	0.43	0.70
15%	Left-Hinge	0.13	**0.85**	0.06	**0.85**	0.50	**0.91**	0.21	0.96	0.23	0.81	**0.07**	0.71
	Right-Hinge	0.15	0.83	0.08	0.81	0.52	**0.91**	0.21	**0.97**	**0.17**	**0.84**	0.14	0.69
	UIM	0.16	**0.85**	**0.048**	0.84	**0.084**	0.84	**0.011**	0.77	0.19	0.83	0.102	**0.90**
	Pix2Pix GAN	**0.09**	0.80	0.11	0.67	0.09	0.80	0.016	0.84	0.22	0.74	0.40	0.69
20%	Left-Hinge	**0.08**	0.84	**0.05**	**0.85**	0.46	0.89	0.18	**0.97**	**0.21**	**0.82**	**0.10**	0.68
	Right-Hinge	0.14	0.83	0.08	0.81	0.47	**0.91**	0.18	**0.97**	0.24	0.79	0.16	0.63
	UIM	0.17	**0.86**	**0.05**	0.84	**0.082**	0.84	**0.011**	0.82	**0.21**	**0.82**	0.13	**0.85**
	Pix2Pix GAN	0.09	0.81	0.08	0.77	0.081	0.81	0.016	0.83	0.27	0.79	0.39	0.67

Significant values are in [bold].

In Table 7, we present the evaluation results that demonstrate the robustness of the Hinge-FM2I, when applied to time series data with varying characteristics. Specifically, we consider two types of time series: stationary(no trend no seasonality) and non-stationary. To determine the stationarity of the time series, we employed the Augmented Dickey-Fuller (ADF) test, where the null hypothesis (H0) assumes the presence of a unit root, indicating non-stationarity, while the alternative hypothesis (H1) suggests stationarity. For each time series type, we introduced gaps of varying sizes (5, 10, and 20) within the data and assessed the performance of our method using three evaluation metrics: sMAPE, RMSE, and MAE. Obtained results are reported separately for the left and right hinges, which represent the regions before and after the introduced gaps, respectively. The findings highlight the effectiveness and robustness of Hinge-FM2I in handling gaps within univariate time series data, as evidenced by the reasonably low error values across different gap sizes and time series characteristics, regardless of their stationarity.

**Table 7:** Comparison of the robustness of our approach on time series with various features.

Time series feature	Gap size	Left-Hinge			Right-Hinge		
		sMAPE	RMSE	MAE	sMAPE	RMSE	MAE
Stationary Time Series	5	9.98	486.63	392.11	9.59	454.72	368.42
	10	13.03	895.84	643.79	13.36	883.69	642.54
	20	16.22	1024.57	773.52	15.92	1051.11	778.59
Non-Stationary Time Series	5	5.95	277.04	226.63	5.72	270.50	219.10
	10	8.33	400.26	322.74	8.75	434.45	346.52
	20	10.31	520.02	415.18	10.71	552.09	439.87

**Figure 5:**
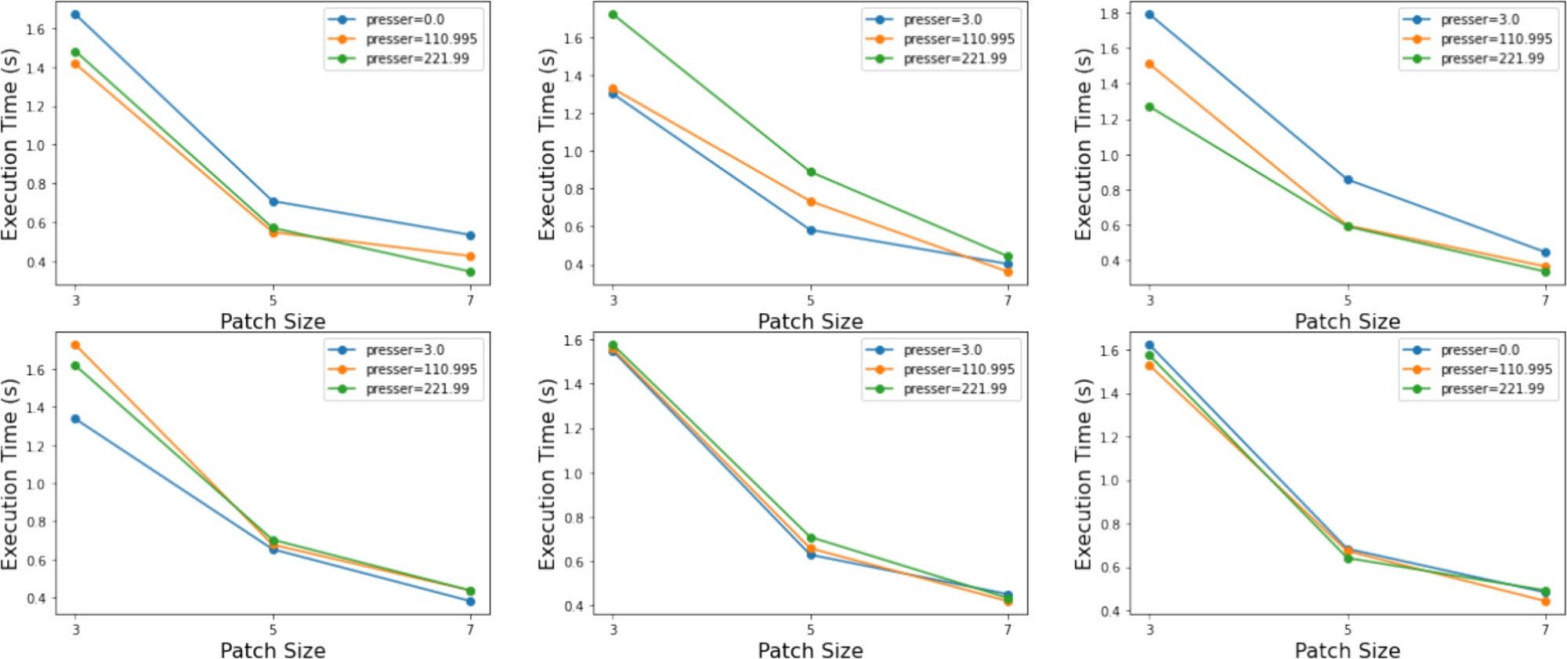
Computational times based on patch size for six different images transformation and their inpainting.

Alongside evaluating the imputation accuracy of our proposed method, we further analyzed its computational efficiency across different configurations. As shown in Table 8, it reports the minimum, maximum, and mean execution times measured over 10 independent runs, while considering variations in matrices, patch sizes, and presser. In order to provide deeper insights, we also showcase, in Fig. 5, different computational time of various transformation and their inpainting as well. We can see that using a higher patch size gives a decent computational time.

**Table 8:** Comparison of computational time of the proposed approach.

Method	Gap of size 5			Ggap of size 10			Gap of size 20		
	Min	Mean	Max	Min	Mean	Max	Min	Mean	Max
Left_hinge	0.49	1.25	3.29	0.72	1.79	4.02	1.37	3.65	8.86
Right_hinge	0.57	1.21	2.72	0.84	1.94	6.07	1.32	3.21	7.04

## Conclusions and perspectives

This study addressed the challenging problem of imputing large gaps of missing data in univariate time series. We proposed a novel method, Hinge-FM2I, which build upon the existing FM2I approach to select optimal replacements for large gaps of missing data. Extensive evaluations demonstrated the effectiveness of our proposed methods over widely-used imputation techniques across various metrics, highlighting its ability in choosing replacements. While the findings presented in this work offer valuable insights, opportunities for further research exploration remain. One potential direction is the investigation of the Both Hinge-FM2I method, taking into consideration both sides of the missing gap, rather than relying solely on data from one end. Additionally, we could explore strategies for selecting diverse elements from multiple rows, instead of utilizing consecutive points from a single row. The substantial improvements in imputation accuracy achieved by Hinge-FM2I are highlighted in Table 9. For instance, when imputing a gap of size 5, our method achieved a sMAPE of 6.4, significantly outperforming the worst possible choice sMAPE = 197.2 and even approaching the theoretically ideal but often impractical best choice sMAPE = 0.99. However, the current implementation of Hinge-FM2I faces several important limitations being addressed in our ongoing and future work. For instance, the computational requirements of the image transformation and inpainting processes make real-time implementation challenging, particularly in resource-constrained environments (e.g., limited memory and processing power). In fact, the algorithm’s execution time increases with larger datasets, which may limit its applicability for time-critical applications (e.g., Curve speed warning).

**Table 9:** Comparison of forecast accuracy metrics.

Size	Worst choice	Hinge choice	Best choice
5	197.2	6.4	0.99
10	196.33	10.57	0.62
20	196.91	12.41	1.12

Several promising directions are identified for further improvement of the Hinge-FM2I. One potential direction is the investigation of the method when using both sides of the missing gap, we named it Both Hinge-FM2I. Rather than relying solely on data from one side, Both Hinge-FM2I handles missing data from left and right sides of the gap, aiming to enhance the accuracy. We could also explore strategies for selecting diverse elements from multiple rows, instead of utilizing consecutive points from a single row. Furthermore, a patch-based algorithm^43^ has shown promising results for image restoration and interpolation and is being investigated for integration into Hinge-FM2I. Future work should also focus on developing optimized implementations specifically designed for embedded systems, potentially through algorithm simplification or the use of hardware acceleration techniques. In fact, the computational efficiency could be enhanced through parallelization of matrix generation and inpainting processes, making the method more suitable for real-time applications.

## Data Availability

The first datasets used in this study are available in the cited publication^25^. The $NH_4$ dataset can be accessed through the R package imputeTS, available at https://github.com/SteffenMoritz/imputeTS. The wastewater treatment plant dataset is publicly available in the University of California at Irvine machine learning repository (UCI) https://archive.ics.uci.edu/.
